# Enriched expression of *NF1* in inhibitory neurons in both mouse and human brain

**DOI:** 10.1186/s13041-019-0481-0

**Published:** 2019-06-24

**Authors:** Hyun-Hee Ryu, Minkyung Kang, Jinsil Park, Sung-Hye Park, Yong-Seok Lee

**Affiliations:** 1 Department of Physiology, Seoul National University College of Medicine, Seoul, 03080 Korea; 2 Department of Life Science, Chung-Ang University, Seoul, 06974 Korea; 3 Department of Biomedical Sciences, Seoul National University College of Medicine, Seoul, 03080 Korea; 4 Department of Pathology, Seoul National University Hospital, Seoul, 03080 Korea; 5 Neuroscience Research Institute, Seoul National University College of Medicine, Seoul, 03080 Korea

**Keywords:** Neurofibromatosis type 1, Neurofibromin, Inhibitory neurons, RAS

## Abstract

Neurofibromatosis type 1 (NF1) is an autosomal dominant disease caused by loss-of-function mutations in *NF1* gene, which encodes a GTPase activating protein for RAS. NF1 affects multiple systems including brain and is highly associated with cognitive deficits such as learning difficulties and attention deficits. Previous studies have suggested that GABAergic inhibitory neuron is the cell type primarily responsible for the learning deficits in mouse models of NF1. However, it is not clear how NF1 mutations selectively affect inhibitory neurons in the central nervous system. In this study, we show that the expression level of *Nf1* is significantly higher in inhibitory neurons than in excitatory neurons in mouse hippocampus and cortex by using in situ hybridization. Furthermore, we also found that *NF1* is enriched in inhibitory neurons in the human cortex, confirming that the differential expressions of *NF1* between two cell types are evolutionarily conserved. Our results suggest that the enriched expression of *NF1* in inhibitory neurons may underlie inhibitory neuron-specific deficits in NF1.

**Electronic supplementary material:**

The online version of this article (10.1186/s13041-019-0481-0) contains supplementary material, which is available to authorized users.

Neurofibromatosis type 1 (NF1) is an autosomal dominant disorder caused by loss of function mutations in *NF1* gene, which occurs in approximately 1 of 3000 births [1]. NF1 affects multiple organs, mainly skin, bone, and brain, and is diagnosed by café-au-lait spots, neurofibromas, optic glioma, Lisch nodules in iris, bone malformations [1–3]. *NF1* is most abundantly expressed in the nervous system [4]. Subsequently, a wide range of cognitive deficits is associated with NF1, which include deficits in visuospatial perception, executive functioning, attention, social function and learning [5–7]. *NF1* gene encodes neurofibromin (NF1) which is a GTPase-activating protein (GAP) for RAS [8–10]. Thus, loss of function mutations in *NF1* gene cause increases in the activation of RAS and its downstream signaling cascades [11]. Studies using mouse models of NF1 have shown that the enhanced activation of RAS-extracellular signal-related kinase (ERK) signaling is responsible for the learning deficits in NF1 [11–14]. *Nf1* heterozygous knockout mice showed deficits in spatial learning and working memory, which can be rescued by attenuating RAS activation [12, 14]. Interestingly, elegant studies by Silva and colleagues have shown that gamma-aminobutyric acidergic (GABAergic) inhibitory synaptic function is altered in both hippocampus and cortex of *Nf1*^+/−^ mice [12, 13, 15]. To define the cell type responsible for the learning deficits in *Nf1*^+/−^ mice, Cui and colleagues deleted *Nf1* selectively in excitatory neurons, inhibitory neurons, or glia and found that deleting *Nf1* only in inhibitory neurons can recapitulate behavioral and cellular phenotypes shown in *Nf1*^+/−^ mice such as deficits in spatial learning and long-term synaptic plasticity [13]. Since NF1 was shown to be ubiquitously expressed in adult neurons, oligodendrocytes, and Schwann cells [4, 16], it is intriguing that deleting *Nf1* selectively affect inhibitory neurons. Recently, we have shown that the genes in RAS-ERK signaling network are differentially expressed between excitatory and inhibitory neurons in mouse hippocampus by performing cell type-specific transcriptome analyses [17]. Interestingly, *Nf1* expression was found to be higher in vesicular gamma-aminobutyric acid transporter (vGAT)-positive neurons than in alpha Ca^2+^/calmodulin-dependent kinase II (αCaMKII)-positive neurons in mouse hippocampus by using cell type-specific RNA-sequencing (RNA-seq) analysis [17], which suggest that inhibitory neuron-enriched expression of *NF1* may underlie the cell type-specific pathophysiology of NF1.

To confirm the expression pattern of *Nf1* in mouse brain (male C57Bl/6 J, 7–8 weeks) by using a different method, we performed fluorescent in situ hybridization. We used a gene-specific probe for mouse *Nf1* together with probes for *αCamkII* and *vGAT* as markers for excitatory and inhibitory neurons, respectively. Consistent with the previous RNA-seq result [17], we found that the *Nf1* expression level is significantly higher in inhibitory neurons than in excitatory neurons in the mouse hippocampus (Fig. 1a and b). The area of *Nf1* mRNA particles in *vGAT*^+^ neurons were significantly larger than in *αCamkII*^+^ neurons in hippocampal CA1 region (Area of *Nf1* particles: *αCamkII*^+^, 3.97 ± 0.16 μm^2^; *vGAT*^+^, 8.25 ± 1.24 μm^2^; unpaired t-test, ****p < 0.0001; Fig. 1a and b). Next, we examined the expression of *Nf1* in mouse cortex (Fig. 1c and d). As in the hippocampus, total area of *Nf1* mRNA particles were bigger in *vGAT*^+^ neurons than in *αCamKII*^+^ neurons in the parietal cortex (Area of *Nf1* particles: *αCamkII*^+^, 3.21 ± 0.21 μm^2^; *vGAT*^+^, 6.1 ± 0.46 μm^2^; unpaired t-test, ****p < 0.0001; Fig. 1c and d). Thus, these results show that the *Nf1* is enriched in *vGAT*^+^ inhibitory neurons in the mouse hippocampus and cortex, which are hubs of spatial learning and higher-level executive brain function. This inhibitory neuron-enriched expression of *Nf1* might explain how inhibitory synaptic function is selectively affected in *Nf1* mutant mice.

**Fig. 1:**
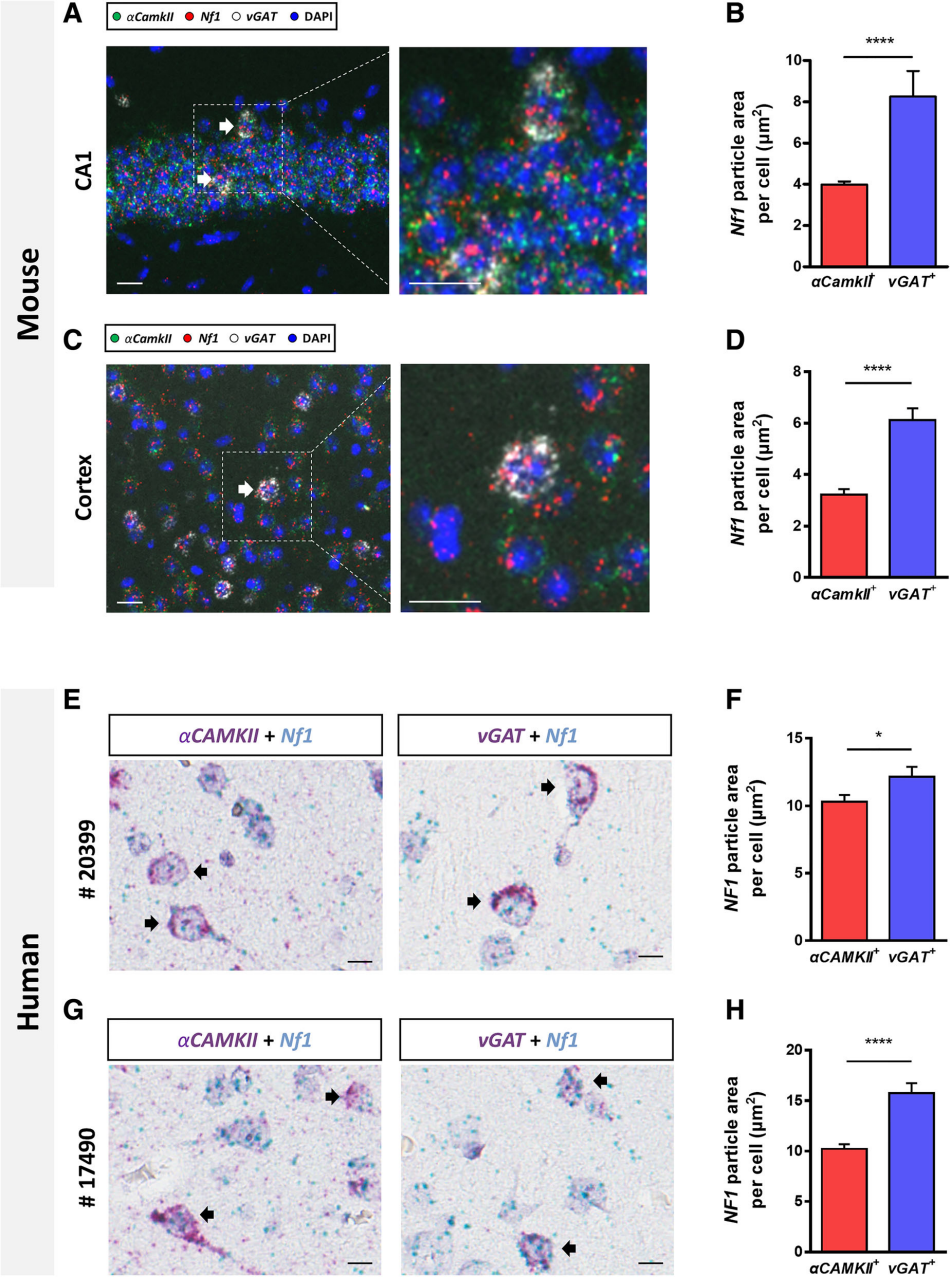
In situ hybridization of *Nf1* in mouse and human brain**. a** Representative merged image of triple fluorescent in situ hybridization probed for *Nf1* (red), *αCamkII* (green) and *vGAT* (white) in hippocampal CA1 region. Higher-magnification images of the boxed area in (**a**) were also shown. White arrows indicate double-positive cells for *Nf1* and *vGAT*. Sections were also stained with DAPI (blue). In situ hybridization was performed following the manufacturers’ manual (RNAscope Multiplex Fluorescent Reagent Kit, Advanced Cell Diagnostics) and the following probes (Advanced Cell Diagnostics) were used: mouse *Nf1*, catalog #417351; mouse *Slc32a1*-C2, #319191-C2; mouse *CamK2*-C3, #445231-C3. Images were acquired by using Axio scan Z1 (Zeiss) and analyzed by using ImageJ (NIH). Scale bar, 10 μm. **b** Average particle size in *αCamk*II^+^ neurons or *vGAT*^+^ neurons in CA1. Data were collected from 627 *αCam*kII^+^ cells and 76 *vGAT*^+^ cells in hippocampal CA1 area. **c** Representative merged image of triple fluorescent in situ hybridization probed for *Nf1* (red), *αCamkII* (green) and *vGAT* (white) in the perietal cortex. Scale bar, 10 μm. **d** Average particle size in *αCaMKII*^+^ neurons or *vGAT*^+^ neurons in the cortex. Data were collected from 127 *αCamkII*^+^ cells and 74 *vGAT*^+^. Data is expressed as means ± SEM. Unpaired t-test, ****p < 0.0001. **e, g** Representative merged image of duplex chromogenic in situ hybridization probed for *NF1* (blue) and *αCaMKII* (red) or *NF1* (blue), *vGAT* (red) and hematoxylin for counter-staining (light-purple color) in human cortex [**e**, sample #20399, 3 years old female diagnosed with focal cortical dysplasia type I (temporal cortex); **g** sample #17490, 2 years old male diagnosed with focal cortical dysplasia type I (frontal cortex)]. Black arrows indicate co-stained cells for either *NF1* and *αCaMKII* or *NF1* and *vGAT*. Chromogenic detection methods according to the manufacture’s instruction (RNAscope duplex chromogenic detection Kit, Advanced Cell Diagnostics). Gene specific probes for human *NF1*, α*CAMKII*, and *vGAT* (human *NF1*, catalog # 419731; human *SLC32A1*-C2, #415681-C2; human *CAMK2*-C2, #521261-C2) were used. Images were acquired by using Aperio scan (Leica Biosystems) and analyzed by using ImageJ (NIH). Images were separated into 3 determined colors (red, green and blue) by ‘colour deconvolution’ plugin which transforms multiple-color images into separated single color channels. Scale bar, 10 μm. **f** and **h** Average *NF1* particle size in *αCaMKII*^+^ neurons or *vGAT*^+^ neurons from #20399 or #17490. Data were collected from 99 *αCaMKII*^+^ cells and 92 *vGAT*^+^ cells in #20399; 142 *αCaMKII*^+^ cells and 98 *vGAT*^+^ cells in #17490. Data is expressed as means ± SEM. Unpaired t-test, *p < 0.01, ***p < 0.001

To verify that *NF1* expression is also higher in inhibitory neurons than in excitatory neurons in human, we examined the *NF1* mRNA expression in human cortex. Since the human tissues showed strong auto-fluorescent signals probably due to the fix condition, we used dual color chromogenic in situ hybridization system: *NF1* was co-stained with either *αCaMKII* (also known as *CaMK2A*) or *vGAT* (also known as *SL32A1*). We used cortical biopsy samples from two human subjects who underwent surgery for focal cortical dysplasia type I. Normal cortical tissues around the affected area were used in this study. As previously reported [18], *NF1* was detected in neurons in human brains (Fig. 1e and g). To examine whether *NF1* is also enriched in inhibitory neurons in human, we analyzed the area of *NF1* mRNA particle in *vGAT*^+^ or *αCaMKII*^+^ neurons. Consistent with our finding in mouse cortex, area of human *NF1* particle in each cell was also significantly larger in *vGAT*^+^ neurons than in *αCaMKII*^+^ neurons in both samples (Area of *NF1* particles: #20399, *αCamkII*^+^, 10.31 ± 0.4 μm^2^; *vGAT*^+^, 12.14 ± 0.74 μm^2^; unpaired t-test, *p < 0.0001; #17490, *αCamkII*^+^, 10.21 ± 0.48 μm^2^; *vGAT*^+^, 15.76 ± 0.98 μm^2^; unpaired t-test, *p < 0.0001; Fig. 1f and h). Collectively, we found that *NF1* mRNA is enriched in *vGAT*^+^ neurons compared to *αCaMKII*^+^ neurons both in mouse and human brain. Importantly, hybridizations using a negative control probe targeting a bacterial gene *dihydrodipicolinate reductase* (*DapB*) did not show any nonspecific background signal in mouse and human cortex (Additional file 1: Figure S1). To further examine the specificity of the probes, we performed cross-species hybridization experiments in which we used the mouse *Nf1* probe on human tissue and the human *NF1* probe on mouse tissue. We detected almost no signals compared to those from species-matching conditions (Additional file 1: Figure S1).

Initially, Costa and colleagues found that monosynaptically evoked inhibitory postsynaptic potentials are significantly larger in *Nf1*^+/−^ mice compared to those in wild type littermates, which was reversed by reducing Ras activity, showing that *Nf1* deletion increases synaptic inhibition through Ras activation [12]. Later, Cui and colleagues showed that neurofibromin regulates ERK and synapsin phosphorylation in GABAergic neurons [13]. However, it is not clear how neurofibromin mainly regulates inhibitory synaptic functions. Recently, Omrani and colleagues showed that neurofibromin interacts with hyperpolarization-activated cyclic nucleotide-gated (HCN) channel which is enriched in parvalbumin (PV)-expressing interneurons [19]. HCN current is selectively reduced in PV-expressing interneurons, resulting in hyperexcitability in PV-expressing inhibitory neurons both in *Nf1*^+/−^ and *Nf1*^*9a−/9a-*^ mice in which the neuron-specific exon 9 is deleted, which may contribute to inhibitory neuron-specific phenotypes in NF1 mouse models [19].

In our previous study, we showed that major components in RAS-ERK signaling pathway including *Nf1* are differentially expressed between excitatory and inhibitory neurons in mouse hippocampus, proposing that this cell type-specific distribution of signaling molecules may underlie cell type selective pathophysiology observed in NF1 and other Rasopathies such as Noonan syndrome [17]. The expression pattern of NF1 has been extensively studied both in rodents and human brains, which have revealed that NF1 is expressed in neurons, oligodendrocytes, and Schwann cells, and even astrocytes depending on the conditions and the isoforms [4, 16, 18, 20–22]. However, it was not clear in which neuronal type *NF1* is enriched. Our results clearly demonstrate that *NF1* is enriched in inhibitory neuron in mouse and human brain. Specific inhibitory neuronal types in which *NF1* is enriched remains to be determined. Taken together, our results strongly suggest that the cell type-specific RAS-ERK signaling networks might be, at least for several molecules, evolutionarily conserved between mouse and human and therefore, the mechanisms for cognitive deficits revealed in NF1 mouse models may turn out to be also true in human NF1.

## Additional file

Additional file 1: **Figure S1.** Representative image of in situ hybridization by using a negative control probe or cross-species probes in human and mouse cortex. (A-B) Human (A) or mouse (B) cortex samples were hybridized with a probe targeting *DapB* by using a fluorescent or chromogenic hybridization method, respectively. No *DapB* signal (blue in A, red in B) was detected. Slices were counter-stained with hematoxylin or DAPI. (C-D) Cross-species hybridization. (C) Human cortex sample was hybridized with the mouse *Nf1* probe (blue). (D) Mouse cortex sample was hybridized with the human *NF1* probe (blue). Slices were counter-stained with hematoxylin. Scale bars, 50 μm. (PDF 131 kb)

## Data Availability

All data generated or analyzed during this study are included in this published article.

## Abbreviations

ERK: Extracellular signal-regulated kinases
GABAergic: Gamma-aminobutyric acidergic
GAP: GTPase-activating protein
HCN: Hyperpolarization-activated cyclic nucleotide-gated channel
NF1: Neurofibromatosis type I
PV: Parvalbumin
RNA-seq: RNA-sequencing
vGAT: Vesicular gamma-aminobutyric acid transporter
αCaMKII: Alpha Ca^2+^/calmodulin-dependent kinase II
