# Supplementary material for: Enriched expression of NF1 in inhibitory neurons in both mouse and human brain
Source: Mol Brain. 2019 Jun 24;12:60. doi: 10.1186/s13041-019-0481-0 (PMC6591896; doi:10.1186/s13041-019-0481-0)
Supplement: Supplementary file 1 — Figure S1. Representative image of in situ hybridization by using a negative control probe or cross-species probes in human and mouse cortex. (A-B) Human (A) or mouse (B) cortex samples were hybridized with a probe targeting DapB by using a fluorescent or chromogenic hybridization method, respectively. No DapB signal (blue in A, red in B) was detected. Slices were counter-stained with hematoxylin or DAPI. (C-D) Cross-species hybridization. (C) Human cortex sample was hybridized with the mouse Nf1 probe (blue). (D) Mouse cortex sample was hybridized with the human NF1 probe (blue). Slices were counter-stained with hematoxylin. Scale bars, 50 μm. (PDF 131 kb) [file 13041_2019_481_MOESM1_ESM.pdf]

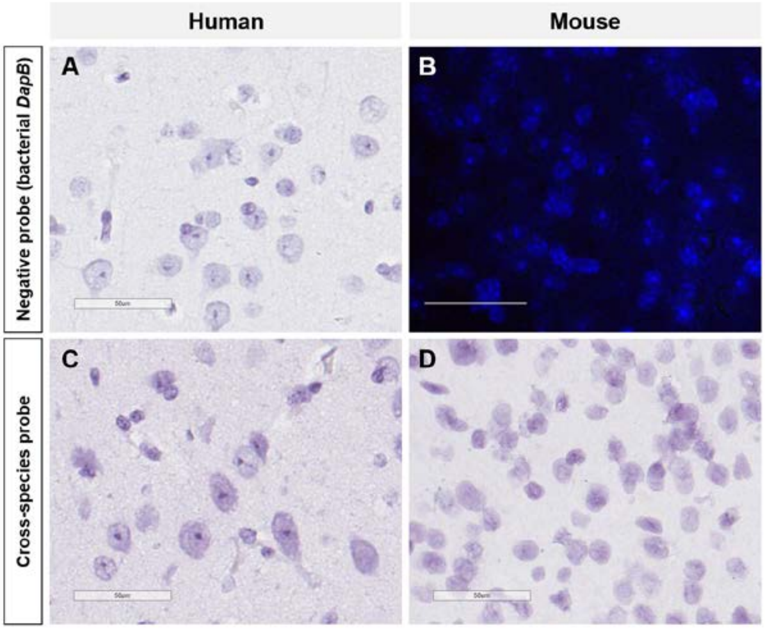

Figure S1. Representative image of *in situ* hybridization by using a negative control probe or cross-species probes in human and mouse cortex. (A-B) Human (A) or mouse (B) cortex samples were hybridized with a probe targeting *DapB* by using a fluorescent or chromogenic hybridization method, respectively. No *DapB* signal (blue in A, red in B) was detected. Slices were counter-stained with hematoxylin or DAPI. (C-D) Cross-species hybridization. (C) Human cortex sample was hybridized with the mouse *Nf1* probe (blue). (D) Mouse cortex sample was hybridized with the human *NF1* probe (blue). Slices were counter-stained with hematoxylin. Scale bars, 50 µm.
